# Aging-Related Metabolic Dysfunction in the Salivary Gland: A Review of the Literature

**DOI:** 10.3390/ijms22115835

**Published:** 2021-05-29

**Authors:** Nguyen Khanh Toan, Sang-Gun Ahn

**Affiliations:** Department of Pathology, School of Dentistry, Chosun University, Gwangju 61452, Korea; nguyenkhanhtoant57@gmail.com

**Keywords:** salivary glands, aging, metabolism, saliva, dysfunction

## Abstract

Aging-related salivary dysfunction commonly induces the poor oral health, including decreased saliva flow and dental caries. Although the clinical significance of the salivary glands is well-known, the complex metabolic pathways contributing to the aging-dysfunction process are only beginning to be uncovered. Here, we provide a comprehensive overview of the metabolic changes in aging-mediated salivary gland dysfunction as a key aspect of oral physiology. Several metabolic neuropeptides or hormones are involved in causing or contributing to salivary gland dysfunction, including hyposalivation and age-related diseases. Thus, aging-related metabolism holds promise for early diagnosis, increased choice of therapy and the identification of new metabolic pathways that could potentially be targeted in salivary gland dysfunction.

## 1. Introduction

The salivary glands are essential structures of the mouth, with the main role of secreting saliva. There are three major salivary glands (submandibular, sublingual, and parotid) that secrete approximately 95% of saliva, while the remaining 5% is secreted by minor salivary glands. Saliva is secreted by acinar cells, which are categorized into mucous and serous acinar cells in the salivary gland. Mucous cells secrete viscous mucin in the vacuoles, while serous cells secrete water and enzymes. Most of the acinar cells in parotid glands are serous, while those of the sublingual and minor glands are mucous. In the submandibular gland, 10% of acinar cells are mucous, and 90% are serous.

Aging is the gradual decline in body function that affects almost all living organisms, and salivary glands are significantly affected. One of the central events of the aging process is metabolic alteration, which is receiving much attention with the increase in metabolomics studies. These analyses focus on metabolites—the intermediates and the final products of every metabolic reaction—which could illustrate a better understanding of the mechanism and progression of aging.

The aim of this review is to discuss aging-related metabolic alterations in the salivary gland and salivary gland function and highlight some medical applications to rescue aged salivary gland dysfunction.

## 2. Aging and Salivary Gland Degeneration

### 2.1. Structural Change

The relationship between aging and the deterioration of salivary gland structure has been studied extensively. Histological analysis demonstrated that with age, the mean volume of acini declined by approximately 30% in the submandibular glands, nearly 25% in the labial salivary glands, and approximately 12% in the parotid glands. On the other hand, there was a gradual increase in lipid droplet infiltration in the salivary glands, as well as an increase in fibrotic tissue. Moreover, in the submandibular glands, age-related acinar degeneration is accompanied by ductal dilation. In the submandibular glands, there was an increase of 80% in the mean proportion of extralobular ducts, a steep decline in the mean volume of the striated duct, from 60% to 40% of the total duct volume, and a significant increase in that of the nonstriated ducts [1]. These studies confirmed the aging-associated degeneration in the parenchyma structures of the salivary gland, which may impair salivary gland function. In addition to histological alterations, aging also causes numerous modifications in the body, which can be attributed to salivary gland dysfunction, such as a decrease in the number of receptors, which can severely reduce the intensity of stimulation to the salivary gland. Reduced blood flow, impaired neuronal transmission, age-related conditions, and the use of medications in the elderly population can also hamper the function of the salivary glands [2].

### 2.2. Saliva Composition

In addition to structural deterioration, the composition of saliva also changed markedly during aging. Saliva is an acidic mixture with a pH range of 6–7 that contains mainly water (99.5%), proteins, mucins, enzymes, and electrolytes [3]. Previously, several studies suggested changes in saliva composition between healthy elderly and young individuals (Table 1). However, there are some conflicts among these studies. While Nagler and Hershkovich reported that the concentration of inorganic materials (K^+^, Cl^−^, P, and Ca^2+^) increased in older individuals, which can be attributed to the reduction in saliva flow rate, Nassar et al. reported that the amount of Ca^2+^ decreased [4,5]. The differences between healthy and disease-affected participants of the selected age may be the cause of this difference in saliva composition. Specifically, the reduction in salivary antioxidants and immunoglobulins induces salivary gland dysfunction or damage and diseases, including cancers. Recently, Maciejczyk et al. reported that antioxidant enzymes in saliva, including peroxidase, glutathione peroxidase, and catalase, decreased with age [6]. Additionally, several papers have reported that mucin levels (MUC1, MUC2, and MUC7) are reduced significantly in the aged adult group [7,8,9]. Losing mucins increases the chance of inflammation and oral diseases, including burning mouth syndrome and cancers [10,11]. These findings displayed a correlation with the aforementioned salivary gland’s histological and physiological degeneration.

### 2.3. Salivary Flow Rate

Elderly people are more vulnerable to diseases, and there are many diseases that can modify the salivary flow rate, including diabetes mellitus, Sjögren’s syndrome, Alzheimer’s disease, and Parkinson’s disease [12,13,14]. Notably, more than 400 medications are linked with the reduced salivary flow [15]. In addition to these diseases and medications, comparisons between healthy participants and elderly people yield controversial results on the topic of salivary gland function and saliva flow rate.

Many studies indicate degenerative alterations in the histological structure of salivary glands with age. This suggests that there may be an age-related functional reduction in saliva flow rate. However, based on a 3-year-long longitudinal study on salivary flow rate with healthy candidates who did not use any medications, these studies reported that there were no significant reductions in salivary gland function or salivary flow rate [16]. On the other hand, a decrease in the salivary flow rate was reported according to a study of 540 elderly healthy individuals [17]. In this study, there was a significant decrease in the whole salivary flow rate and the submandibular and sublingual glands’ salivary flow rate of elderly candidates under both resting and stimulated conditions. Another review showed that the resting salivary flow rate decreased by 44% in older participants, while that of stimulated participants was 15%. The resting submandibular and sublingual glands flow rate was reduced by 11%, and the stimulated submandibular and sublingual glands flow rate was decreased by 9%. On the other hand, the differences in the parotid gland and minor gland salivary flow rates are not significant [18]. A summary of aging-induced changes is shown in Table 1.

## 3. Metabolic Changes in Salivary Gland

The metabolic processes of biological systems are influenced by the genomics, transcriptomics, proteomics, environmental alterations, and pathophysiological and developmental conditions of that specific biological system [21]. Because aging is a complex process that is influenced by a combination of genetics, the environment, diet, and lifestyles, metabolomics are becoming powerful tools to analyze the myriad of interactions and generate profiles of aging-related alterations in the body, thus providing better information about novel pathways and biomarkers and improving clinical approaches [22]. In the narrow field of salivary gland-related metabolomics studies, the majority of these studies focused on the discovery of disease biomarkers, from salivary gland-related diseases such as Sjögren’s syndrome to oral and periodontal diseases and to neurodegenerative diseases such as dementia, Alzheimer’s disease, and even cancers [23,24,25,26,27,28,29,30,31,32]. In Sjögren’s syndrome, metabolomics research using nuclear magnetic resonance (NMR) revealed that the concentrations of choline, taurine, alanine, glycine, butyrate, phenylalanine, and proline increased significantly in the saliva samples of Sjögren’s syndrome patients compared with healthy candidates. Notably, the lower salivary flow rate in Sjögren’s syndrome patients is correlated with higher concentrations of choline and taurine, suggesting that decreasing saliva as a solvent may lead to an increase in these metabolites [33]. Another study using mass spectrometry (MS) demonstrated that the diversity of the salivary metabolome heavily impacted Sjögren’s syndrome patients, with 41 metabolites found to be reduced, which were mainly amino acids and carbohydrates [24]. A large-scale study conducted in 2019 with over 900 candidates demonstrated that the metabolite phenylacetate, which is a product of fermentation by oral bacteria, is a novel biomarker for periodontitis. In particular, the concentration of phenylacetate is positively associated with periodontal pocket depth in all age groups [25]. In oral squamous cell carcinoma patients, the levels of two metabolites, namely, glycine and proline, are significantly reduced compared to those in normal control candidates, as confirmed by both NMR and MS methods [34]. A systematic review conducted by Assad et al. summarized that the combination of choline, betaine, pipecolinic acid, and L-carnitine provided outstanding sensitivity and specificity for diagnosing oral cancers [35]. Recently, there have been two large-scale studies on the salivary metabolome to identify novel markers for Alzheimer’s disease (474 and 1246 candidates, respectively), which illustrated that sphinganine-1-phosphate, an intermediate of glycosphingolipid and sphingolipid metabolism, as well as a substrate of sphingosine kinase, is upregulated in Alzheimer’s disease patients [31,36]. These studies clearly demonstrated the potential of metabolomics to provide key elements for further study concerning the disease’s mechanism and early diagnosis.

Recently, we conducted a series of studies to evaluate aging-induced salivary gland dysfunction in an accelerated-aging mouse model generated by crossing klotho mutants and SAMP1 mice. These mice had a shorter average lifespan (9 weeks), lower average body weight, and developed extensive tissue inflammation and calcification [37]. Using capillary electrophoresis time-of-flight mass spectrometry (CE-TOF/MS) to analyze the metabolome of aging mice, we detected 232 metabolites (134 metabolites in cation mode and 98 metabolites in anion mode) based on the HMT standard library. Specifically, we found that the aged salivary gland leads to a systemic alteration of numerous metabolic pathways, including glycolysis/gluconeogenesis, the pentose-phosphate pathway, the tricarboxylic acid cycle, the urea cycle, nucleotide metabolism, glutathione metabolism, and acetylcholine metabolism [38]. Importantly, in the salivary gland of aging mice, aging induces oxidative stress with the reduction in numerous antioxidant metabolites, such as carnosine, ergothioneine, and glutathione. A summary of all the metabolite changes is presented in Supplementary Table S1.

### 3.1. Innervation of Salivary Gland

The salivary glands are controlled by both the sympathetic and parasympathetic nervous systems. More specifically, parasympathetic stimulation leads to water and ion secretion, while sympathetic stimulation leads to the secretion of proteins [39].

Sympathetic innervation of the salivary glands starts with the preganglionic nerves in the thoracic segment of the spinal cord. These nerves clustered from the thoracic ganglion to the superior cervical ganglion and then spread parallel to the carotid artery. From the carotid plexus, these nerves branch out and innervate the organs along the facial blood vessels [40,41].

**Figure 1:**
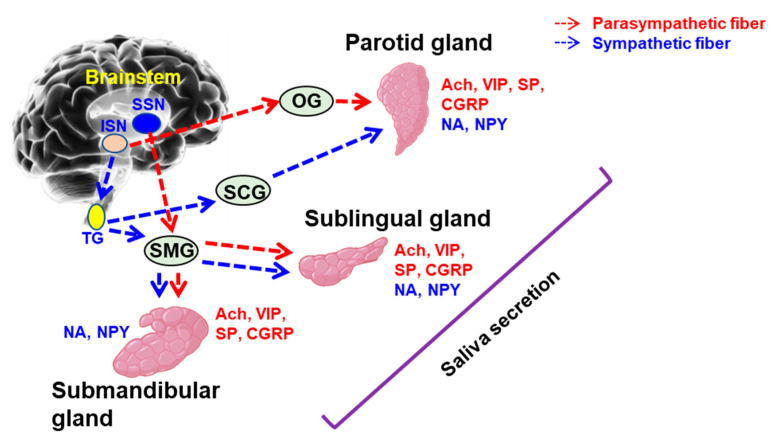
Salivary gland innervation. SSN: superior salivary nucleus; ISN: inferior salivary nucleus; TG: thoracic ganglion; SMG: submandibular ganglion; SCG: superior cervical ganglion; OG: otic ganglion; Ach: acetylcholine; VIP: vasoactive intestinal peptide; SP: substance P; CGRP: calcitonin gene-related peptide; NA: neurokinin A; NPY: neuropeptide Y.

Parasympathetic innervation occurs from the salivatory nuclei located in the brainstem. From there, the facial nerve (CN VII) innervates the submandibular and sublingual salivary gland, while the glossopharyngeal nerve (CN IX) innervates the parotid gland [41,42]. The salivary gland innervation is illustrated in Figure 1.

### 3.2. Neurochemical Metabolites of Salivary Gland

Saliva secretion, the main function of the salivary gland, is controlled by neural regulation through several neurotransmitters. Numerous stimulations can evoke saliva secretion, including taste, smell, temperature, and chemicals. These stimuli trigger the salivatory nucleus, causing a wave of sympathetic and parasympathetic signals to the salivary glands. Saliva secretion is then modulated by both sympathetic and parasympathetic signaling.

Sympathetic signaling is modulated by norepinephrine, which binds to and activates adrenergic receptors (α1 and β1 in the salivary glands) [39,43]. On the other hand, parasympathetic signaling revolves around acetylcholine and several nonnoradrenergic, noncholinergic (NANC) transmitters, such as vasoactive intestinal peptide (VIP) or neuropeptide Y (NPY), neurokinin A (NKA), substance P (SP), pituitary adenylate cyclase activating peptide (PACAP), neuronal nitric oxide synthase (nNOS), and calcitonin gene-related peptide (CGRP) [39]. In the salivary gland, acetylcholine interacts with muscarinic cholinergic receptors (mAchR – mainly M1 and M3) to stimulate salivary secretion. Acetylcholine and norepinephrine induce saliva secretion by activating the M1 and M3 receptors. Following the activation of M1 and M3 receptors, inositol triphosphate (IP3) is generated and then binds to the IP3 receptors on the surface of the endoplasmic reticulum, which triggers the release of intracellular Ca^2+^. The elevated level of intracellular Ca^2+^ opens chloride and potassium ion channels on the membrane, leading to electrolyte and water secretion [43]. Norepinephrine and VIP activate β1 adrenergic receptors and induce the cyclic adenosine monophosphate/protein kinase A (cAMP/PKA) signaling pathway, leading to salivary protein secretion [44].

The NANC transmitters also contribute to saliva secretion. PACAP can enhance salivary secretion by binding to its receptors in three major salivary glands [45]. Tachykinins, such as NKA and SP, can also induce saliva secretion through intracellular Ca^2+^ signaling, most likely through the tachykinin receptor NK1 [46,47,48]. Both neuronal and endothelial nitric oxide synthase produce the free radical nitric oxide, which can influence saliva and protein secretion [49]. CGRP can modulate voltage-dependent calcium channels, one of the key regulators of calcium influx, which are involved in saliva secretion [50]. A summary of the role of several neurochemicals is presented in Table 2.

Although neuropeptides contribute largely to the regulation of saliva secretion, recent studies illustrated that parasympathetic denervation leads to an increase in the expression of salivary gland functional markers and resting saliva secretion in a long-term manner [51,52]. These data highlight the regenerative capability of the salivary gland through autonomic reinnervation.

Moreover, evidence has shown that the nervous system is also involved in the development of the salivary glands. The innervation of the salivary gland starts during the embryonic stage and progresses with the organogenesis of the salivary gland. In mice, at day E14, the salivary gland is fully branched and densely innervated [53]. Removal of the parasympathetic submandibular ganglion ablates the branching of salivary glands and reduces the expression of epithelial markers. Acetylcholine, which is secreted by parasympathetic nerves, maintains the stemness of epithelial salivary gland stem cells during organogenesis via the muscarinic M1 receptor and EGFR [54]. Targeting the nervous system may induce salivary gland regeneration, for example, through the growth factor neurturin or through the pharmaceutical agonists of muscarinic and adrenergic receptors [55,56].

Recently, we published a study reporting that klotho depletion impaired acetylcholine metabolism through inhibition of the synthetic enzyme choline acetyltransferase (ChAT) [38]. ChAT is the enzyme that catalyzes the biosynthesis of acetylcholine from choline and acetyl-CoA. Depletion of Ach leads to a reduction in cholinergic signaling in the salivary glands. In vitro and in vivo restoration of ChAT levels rejuvenates salivary gland function, improving salivary gland functional markers, thus providing a novel approach for salivary gland regeneration treatment.

In the same study, they also found a significant reduction in the level of essential amino acids, suggesting a dysregulation of amino acid metabolism in the salivary gland of an aging mouse model. Furthermore, in addition to acetylcholine, several nervous system-related metabolites also have noteworthy alterations, such as histamine, adenosine, and cytidine diphosphate-choline (CDP-choline) [38]. Histamine is a neurotransmitter involved in the inflammatory response and is produced by mast cells and basophils in the tissue surrounding inflammatory sites [59]. In our data, the level of histamine increased markedly in an accelerated-aging mouse model, while the level of histidine, the main precursor of histamine, was reduced in a time-dependent manner. One possibility is that the activity of the catalytic enzyme histidine decarboxylase is increased in inflammatory tissues, since aging mice have widespread oxidative stress-induced inflammation [37,60].

Interestingly, another neurotransmitter, adenosine, also increased in the salivary gland of an accelerated-aging mouse model. Adenosine is an important keystone for every living organism, as it is one of the four building blocks of DNA and RNA. During aging, adenosine is capable of protecting cells and organs in multiple pathological states, such as epilepsy, ischemia, inflammation, autoimmune diseases, and pain [61,62,63,64,65,66,67,68,69], which could be interpreted as a defense mechanism of aging against inflammation and oxidative stress. On the other hand, damage and stress-induced production of adenosine can be easily turned into chronic overproduction of adenosine and is linked with organ damage, fibrosis, and chronic inflammation [70]. Further studies are required to elucidate the role of adenosine accumulation in aged mice.

In addition, CDP-choline, an intermediate metabolite in the biosynthesis of phosphatidylcholine, was also reduced in an accelerated-aging mouse model [38]. CDP-choline exhibits multiple beneficial effects, including neuroprotective, neuroregenerative, and antioxidative stress abilities [71,72,73]. The effectiveness of CDP-choline covers a wide range of neurological diseases, including neurodegenerative diseases; cognitive, emotional, and behavioral disorders; and cerebrovascular disease [74]. It is well developed that aging increases the vulnerability and fragility of the nervous system, and we previously suggested that accelerated-aging mice had impaired acetylcholine biosynthesis, which may indicate aging-induced injury in the innervated nerves of the salivary gland [38]. Losing CDP-choline may further hinder the regenerative ability of the body against aging-induced damage to the nervous system in the salivary gland.

## 4. Endocrine Metabolites of the Salivary Glands

Aging is also accompanied by significant changes in the secretory patterns relevant to the sensitivity of the endocrine axis in the salivary gland [75]. Age-related hormone changes in the salivary gland have a multitude of impacts, both beneficial and detrimental. We summarized the age-related alterations of several hormones and their impact on the salivary glands (Table 3).

### 4.1. Insulin

The hormone insulin, produced by β cells in the pancreas, is the main hormone regulating the metabolism of carbohydrates, fats, and protein. Studies have reported that precursors of insulin and preproinsulin were found in the salivary glands of rats and mice [76,77], which also supports the local synthesis of insulin in the salivary glands. In addition to the metabolic regulation function, is there any relationship between insulin and salivary gland function? It was suggested that insulin in saliva may enhance salty taste sensitivity in mice [78]. Apparently, diabetes patients are more likely to develop xerostomia than non-diabetes patients [79]. On the other hand, patients who develop salivary gland adenoid cystic carcinoma tend to have higher expression of IGF-IR signaling [80], and inhibition of IGF/IR can lead to a reduction in the aggressiveness of the salivary gland cancer cells [81]. Systemic disruption of insulin production by feeding alloxan to rats led to a retardation of body and salivary gland growth [82]. In the diabetic db/db mouse model, which has a higher insulin concentration but also has insulin resistance, the submandibular gland has acinar enlargement, ductal atrophy, mitochondrial dysfunction, and mitophagy compared with normal mice [83]. Additionally, higher oxidative stress and oxidative lipid product accumulation were recorded in the parotid gland of high-fat diet-induced diabetic mice, which also have insulin resistance [84]. These studies indicate that systemic damage induced by a lack of insulin can cause serious damage to the salivary glands.

### 4.2. Melatonin

Melatonin is a hormone that is mostly known as the regulator of the sleep-wake cycle. However, melatonin also expresses antioxidative, antimicrobial, and immunomodulatory effects in the oral cavity and salivary glands [85,86,87,88]. It was found that the salivary glands are capable of synthesizing melatonin through catalytic enzymes expressed in the epithelial cells of striated ducts [89]. Additionally, it was found that two melatonin receptors were localized in the secretory granules and cytoplasmic vesicles of acinar cells [90]. Furthermore, melatonin not only reacts with the receptors in acinar cells but is also stored inside them for further release [91]. These data illustrated that a portion of salivary melatonin might be produced in the salivary glands. It was reported that the concentration of salivary melatonin is reduced with age, starting in the 40s [92]. Melatonin is a powerful antioxidant and anti-inflammatory hormone that can easily penetrate every cell due to its lipophilic nature [93]. Melatonin exhibits a protective effect, as it can increase cellular activity in the submandibular gland of rats [94] and is involved in histological improvement in diabetic rats by inducing vascular endothelial growth factor [95]. Melatonin can also induce protein/amylase secretion from parotid glands, either through its own receptors on acinar cells or in a NOS-dependent manner [96]. Additionally, melatonin is involved in the developmental process of the salivary gland as a regulator, as melatonin can inhibit the epithelial branching of the salivary gland but does not affect cell proliferation or induce cell apoptosis [97].

### 4.3. Estrogens and Androgens

Estrogen and androgen hormones are steroid hormones that help regulate reproduction in men and women and are secreted by the gonads in the hypothalamus-pituitary-gonadal axis. Normally, most sex hormones are bound to specific proteins, and only 5% of them are unbound, remain active, and can penetrate cell membranes and enter saliva.

It is well established that there is a gradual decrease in both total and free sex hormones with age. The first nationally representative survey in the USA also showed that the concentration of salivary sex hormones is reduced significantly with age [98]. Both estrogen and androgen receptors were found in the oral mucosa and salivary glands, indicating that sex hormones might have some influence on the oral and salivary gland function [99,100]. The loss of estrogen during menopause is linked with the development of oral diseases and salivary gland dysfunction in women [101]. In addition, postmenopausal women might be more susceptible to salivary gland-related disorders, such as xerostomia, Sjögren’s syndrome, and burning mouth syndrome. Elderly women represent 50% of cases of xerostomia, and they are also mainly affected by Sjögren’s syndrome, with the majority of patients being women between 40 and 50 years old [101]. Estrogen can enhance the immune system by inducing the production of antibodies and increasing the infiltration of lymphocytes into the salivary glands [102]. Treatment of estrogen in ovariectomized rats, which lack sex hormones, protects the salivary glands against cell apoptosis, gland atrophy, and mitochondrial defects by regulating the expression of SOD1/SOD2 and caspase-3 [103]. However, the cell-protective effect of estrogen can also backfire, as estrogen can enhance the malignancy of salivary adenoid cystic carcinoma cells [104].

Androgens also participate in the modulation of salivary gland function. The concentration of salivary dehydroepiandrosterone (DHEA) in Sjögren’s syndrome patients is lower than that in healthy participants, and the expression of cysteine-rich secretory protein 3, an androgen-regulating biomarker, is also reduced [105]. Similar to estrogen’s protective ability, DHEA treatment allows the renewal of acinar and salivary gland cells in both human and mouse models [106]. The main targets of androgens are the granular duct cells in the salivary glands [107]. Adrenalectomy in mice led to a significant reduction in salivary gland size, granular duct cells, and duct diameter; however, the reduction is reversible by supplementation with testosterone [107]. Administration of 5α-dehydrotestosterone enhances salivary gland development in mice, as demonstrated by the fact that the treated group has more abundant, fully developed granular cells than the control group [108,109].

## 5. Therapeutic Approach for Aging-Induced Salivary Gland Disorders

Due to the numerous causes that can lead to salivary gland disorders, particularly hyposalivation, it is extremely difficult to cure salivary gland diseases. More importantly, there are no permanent solutions to resolve irreversible damage to the salivary gland. Recently, with the advances in regenerative medicine, a branch of research that aims to restore or establish the normal function of damaged tissues, there have been several advances in the field of salivary gland engineering to counter hyposalivation. In this section, we highlight and review some advances in the therapeutic approach to restore salivary gland function.

In the last 20 years, pharmaceutical applications, mainly sialagogue and artificial saliva, have accounted for approximately 50% of the registered treatment methods for hyposalivation [110]. Since saliva secretion is controlled by neurotransmitters secreted from innervated neurons in the salivary gland, these stimulant medications mainly mimic neuronal signals to induce saliva production and secretion [110]. Two well-known and widely used stimulant medications are pilocarpine and cevimeline. Pilocarpine’s main target is muscarinic receptor 1 (M1), while cevimeline’s main target is muscarinic receptor 3 (M3) [111,112,113,114]. However, due to the ubiquitous expression of these muscarinic receptors in the body, the use of pilocarpine and cevimeline may lead to severe side effects, such as frequent urination, dizziness and sweating, nausea, diarrhea, vasodilation, bronchoconstriction, hypotension, and bradycardia [115,116]. Moreover, these medications can interact with pre-existing conditions, such as uncontrolled asthma, chronic pulmonary disease, or cardiovascular diseases; thus, the range of application is limited [117].

Artificial saliva, generally, is a moisturizer that provides temporary moisture and protective properties for oral structures. Artificial saliva is usually based on carboxymethyl cellulose (CMC), mucins, or xanthan gum. However, when compared with natural saliva, artificial saliva exhibits poorer antimicrobial and antifungal effects [118]. This may lead to the dysregulation of the oral microbiome, which consists of an incredible complex of bacteria, viruses, fungi, and phages [119]. A significant alteration in the balance of the oral microbiome may lead to serious oral health consequences [120]. Therefore, to improve the antimicrobial activity of artificial saliva, nanoparticles could be used to deliver antimicrobial agents at an in vitro scale, which will provide viable options for the development of artificial saliva.

A few metabolite-based therapies have also been developed to ameliorate xerostomia. A topical sialagogue spray containing 1% malic acid already showed significant effectiveness against xerostomia patients [121,122]. Malic acid is an important intermediate metabolite in various metabolic pathways, including the Krebs cycle. Retinoic acid (RA), the active metabolite of vitamin A, can modulate the growth and differentiation of epithelial stem cells by activating the Fgf10/Etv5 signaling network and repressing the Krt5/Krt14 signaling pathway. Its effect can be applied to induce the regeneration of injured salivary gland tissues [123,124]. Coenzyme Q10 is an essential enzyme of the respiratory chain that is responsible for transporting electrons from complex I to complex II and from complex II to complex III [125]. During the respiratory chain, coenzyme Q10 exists in two variants, the oxidized form known as ubiquinone and the reduced form known as ubiquinol [126]. Supplementation with ubiquinol increased the salivary flow rate in human participants in both short-term and long-term treatment [127]. In vitro experiments illustrated that ubiquinol can stimulate ATP production and suppress oxidative stress in salivary gland cells, which could contribute to the improvement in the salivary flow rate [127].

## 6. Conclusions

In this review, we summarized the aging-induced changes in the salivary glands from the structural level to the functional level and the metabolic alterations, especially the neurometabolites in the accelerated-aging salivary glands. Metabolomic analysis is a novel approach and has much potential in elucidating the complex process of living organisms, since metabolite alteration is considered the ultimate response of biological systems to genetic diseases or environmental influences [128]. Currently, salivary metabolites are rising stars in the early diagnosis of a plethora of diseases and are not limited to only oral diseases but also to cancers and neurodegenerative diseases. Furthermore, since the metabolites are deeply involved in the cellular metabolism process, it is possible that these metabolites could be targeted for metabolism-based treatment. Further studies are needed to unveil and to utilize the full capacity of metabolites, not only in the salivary gland but also in other organs.

## Figures and Tables

**Table 1:** Aging-induced histological alteration, change in saliva composition, and salivary flow rate.

Reference	Study Design	N of Candidates	Results
Scott et al., 1987 [19]	Histological analysis of parotid salivary glands from dead people	N = 63	Adipose content, fibrotic tissue, and ductal irregularities increase with age. Proportion of acinar structure declines by 30%.
J. Scott, 1977 [1]	Histological analysis of submandibular salivary glands from dead people	N = 96	Reduction in parenchymal tissue and acinar structure. Percentage of adipose tissue increases. Duct volume also increases due to duct dilatation.
J Scott, 1980 [20]	Histological analysis of labial salivary glands from dead people	N = 70	Acinar atrophy, ductal dilatation and hyperplasia increase with age. Acinar volume decreases while the fibrotic tissue proportion increases.
Nagler and Hershkovich, 2005 [4]	Sialometrical and sialochemical analysis of unstimulated saliva	N = 80	Concentrations of K^+^, Ca^2+^, P, amylase and IgA increase. Total amounts of Na^+^, Ca^2+^, Mg^2+^, IgG, and IgA decrease.
Nassar et al., 2014 [5]	Analysis of unstimulated saliva	N = 40	Salivary flow rate and concentrations of Ca^2+^, collagenase type 1 and MMP-8 decrease.
Maciejczyk et al., 2019 [6]	Redox and antioxidant analysis of both resting and stimulated saliva	N = 90	Salivary peroxidase and catalase decrease while peroxidase increases with age.
Chang et al., 2011 [7]	Mucin and cytokine analysis of stimulated saliva	N = 60	MUC1 levels and salivary flow rate decrease in the old age group.
Pushpass et al., 2019 [9]	Analysis of unstimulated and taste stimulated saliva	N = 56	Salivary flow rate and MUC7 levels are decreased in old age group.
Affoo et al., 2015 [18]	Meta-analysis of previous studies involves salivary flow rate and age	N = 47	Salivary flow rate decreased significantly with aging in every gland.

**Table 2:** Role of neurochemicals in salivary glands.

Name	Function	References
Acetylcholine (Ach)	Invokes water secretion through M1/M3 AchR; maintains the stemness of the epithelial salivary gland stem cells during organogenesis	Proctor, 2016 Knox et al., 2010 [54,57]
Norepinephrine	Invokes protein secretion through β1 adrenergic receptors	Straub et al., 2002 [44]
Vasoactive intestinal peptide (VIP)	Invokes protein secretion through β1 adrenergic receptors	Straub et al., 2002 [44]
Neuropeptide Y (NPY)	Induces protein and ion secretion	Ekstrom et al., 1996 [58]
Neurokinin A (NKA)	Stimulates saliva secretion by manipulating intracellular Ca^2+^ signaling	Qi et al., 2010 [46]
Substance P (SP)	Stimulates saliva secretion through tachykinis receptors NK1	Yu et al., 1983 [48]
Nitric oxide synthase (NOS)	Induces saliva secretion through the free radical nitric oxide	Correia et al., 2010 [49]
Pituitary adenylate cyclase activating peptide (PACAP)	Invokes saliva secretion by binding to its receptor PAC1R; increases the EGF level in saliva.	Matoba et al., 2016 [45]
Calcitonin gene-related peptide (CGRP)	Modulates the voltage-dependent calcium channels; enhances NPY-induced saliva secretion	Endoh et al., 2011. [50]

**Table 3:** Several key endocrine-related metabolites in salivary glands.

Name	Function	References
Insulin	Dysfunction of insulin metabolism can induce acinar enlargement, ductal atrophy, mitochondrial dysfunction, mitophagy, oxidative stress, and oxidative lipid accumulation.	Liu and Lin, 1969 Xiang et al., 2020 [82,83]
Melatonin	Induces protein secretion through melatonin receptors and nitric oxide synthase. Induces cellular activity and regulates the organogenesis of embryonic salivary glands	Aras & Ekstrom, 2008 Ashour, 1998 Obana-Koshino et al., 2015 [94,96,97]
Estrogens	Lack of estrogen is highly associated with the development of salivary gland-related diseases. Ovariectomized rats developed cell apoptosis, gland atrophy, and mitochondrial defects, which are all reversible with estrogen administration. Can induce the production of antibodies and increase the lymphocyte infiltration in salivary glands.	Meurman et al., 2009 Ahmed et al., 1989 Da et al., 2015 [101,102,103]
Androgens	Castrated mice have significantly smaller salivary gland size, granular duct cells and duct diameter. Can induce the development of granular cells in salivary glands. DHEA treatment improves the salivary flow rate and acinar cells in Sjögren’s syndrome patients.	Sato et al., 1981 Kurabuchi, 2006 Kurabuchi and Hosoi, 2009 [107,108,109]

## Data Availability

Not applicable.

## Supplementary Materials

The following are available online at https://www.mdpi.com/article/10.3390/ijms22115835/s1, Table S1: Hierarchical cluster analysis (HCA) and heatmap visualization of metabolic profiles.
