# Supplementary material for: Aging-Related Metabolic Dysfunction in the Salivary Gland: A Review of the Literature
Source: Int J Mol Sci. 2021 May 29;22(11):5835. doi: 10.3390/ijms22115835 (PMC8198609; doi:10.3390/ijms22115835)
Supplement: Supplementary file 1 [file ijms-22-05835-s001.zip › ijms-1245031-supplementary.pdf]

HCA

Line	HMT DB †		Standardized Relative Area §		
	Compound name	KEGG ID	SAMP1/KL+/+	SAMP1/KL-/- 1M	SAMP1/KL-/- 2M
1	Creatinine	C00791	-0.310	-0.808	1.118
2	1-Pyrroline 5-carboxylic acid	C03912	-0.536	-0.618	1.154
3	3-Phenylpropionic acid	C05629	-0.577	-0.577	1.155
4	3-Phosphoglyceric acid	C00197	-0.577	-0.577	1.155
5	3-Amino-2-piperidone		-0.577	-0.577	1.155
6	Urea	C00086	-0.728	-0.412	1.140
7	Choline	C00114	-0.737	-0.401	1.138
8	Adenosine	C00212	-0.749	-0.386	1.136
9	2'-Deoxyguanosine	C00330	-0.858	-0.240	1.098
10	*O*-Acetylcarnitine	C02571	-0.873	-0.218	1.091
11	Glutathione (GSSG)_divalent	C00127	-0.898	-0.179	1.078
12	Thymidine	C00214	-0.902	-0.174	1.075
13	3'-CMP<br>2'-CMP	C05822<br>C03104	-0.905	-0.169	1.074
14	2'-Deoxycytidine	C00881	-0.914	-0.154	1.068
15	Betaine aldehyde_+H_2O	C00576	-0.918	-0.148	1.066
16	cCMP<br>2',3'-cCMP	C00941<br>C02354	-0.927	-0.132	1.060
17	1-Methyl-4-imidazoleacetic acid	C05828	-0.934	-0.120	1.055
18	Histamine	C00388	-0.963	-0.071	1.033
19	ATP	C00002	-0.974	-0.051	1.024
20	Citric acid	C00158	-0.975	-0.048	1.023
21	Guanosine	C00387	-0.995	-0.009	1.005
22	ADP	C00008	-1.005	0.011	0.995
23	*S*-Adenosylhomocysteine	C00021	-1.052	0.115	0.938

24	Inosine	C00294	-1.053	0.115	0.937
25	dAMP	C00360	-1.058	0.129	0.929
26	dCMP	C00239	-1.104	0.260	0.845
27	Ribulose 1,5-diphosphate	C01182	-1.112	0.288	0.824
28	γ-Butyrobetaine	C01181	-1.124	0.333	0.791
29	Cysteinesulfinic acid	C00606	-1.126	0.342	0.784
30	Ethanolamine	C00189	-1.129	0.356	0.773
31	dTMP	C00364	-1.143	0.429	0.714
32	UDP	C00015	-1.144	0.434	0.709
33	Pipecolic acid	C00408	-1.146	0.447	0.698
34	2-Deoxyglucose 6-phosphate	C06369	-1.153	0.528	0.625
35	NAD^+	C00003	-1.155	0.576	0.578
36	2-Hydroxyisobutyric acid		-1.155	0.593	0.562
37	CMP-*N*-acetylneuraminate	C00128	-1.153	0.622	0.531
38	XA0035		-1.152	0.650	0.501
39	Acetyl CoA_divalent	C00024	-1.149	0.671	0.478
40	*myo*-Inositol 1-phosphate<br>*myo*-Inositol 3-phosphate	C01177<br>C04006	-1.149	0.672	0.477
41	2-Hydroxyvaleric acid		-1.149	0.677	0.472
42	Glyceraldehyde 3-phosphate	C00118,C00661	-1.147	0.692	0.454
43	Glutaric acid	C00489	-1.143	0.714	0.429
44	Pyridoxamine 5'-phosphate	C00647	-1.141	0.726	0.415
45	Taurocholic acid	C05122	-1.134	0.756	0.377

46	Ethanolamine phosphate	C00346	-1.126	0.786	0.340
47	*N*-Acetylneuraminic acid	C00270	-1.114	0.821	0.293
48	Diethanolamine	C06772	-1.081	0.891	0.190
49	5-Oxoproline	C01879	-1.059	0.928	0.131
50	Cytidine	C00475	-1.057	0.930	0.127
51	Pantothenic acid	C00864	-1.048	0.943	0.105
52	Uric acid	C00366	-1.047	0.945	0.103
53	Homoserine	C00263	-1.046	0.946	0.100
54	CMP	C00055	-1.041	0.953	0.087
55	Rhein	C10401	-0.979	1.020	-0.042
56	Carnitine	C00318,C00487,C15025	-0.971	1.027	-0.056
57	1-Methylhistamine	C05127	-0.959	1.037	-0.078
58	Homocitrulline	C02427	-0.926	1.061	-0.135
59	Ribose 5-phosphate	C00117	-0.881	1.087	-0.206
60	Thiamine phosphate	C01081	-0.873	1.091	-0.218
61	*O*-Acetylhomoserine<br>2-Aminoadipic acid	C01077<br>C00956	-0.856	1.099	-0.243
62	*myo*-Inositol 2-phosphate		-0.820	1.114	-0.294
63	FAD_divalent	C00016	-0.771	1.130	-0.359
64	Dihydroxyacetone phosphate	C00111	-0.768	1.131	-0.362
65	Betaine	C00719	-0.761	1.133	-0.372
66	Asp	C00049,C00402,C16433	-0.729	1.140	-0.411
67	N^5-Ethylglutamine	C01047	-0.724	1.141	-0.417
68	UDP-glucose<br>UDP-galactose	C00029<br>C00052	-0.657	1.151	-0.494
69	Acetoacetamide	C11106	-0.647	1.152	-0.505

70	CoA_divalent	C00010	-0.639	1.152	-0.514
71	XC0016		-0.577	1.155	-0.577
72	*S*-Lactoylglutathione	C03451	-0.577	1.155	-0.577
73	cAMP	C00575	-0.577	1.155	-0.577
74	Prostaglandin E_2	C00584	-0.577	1.155	-0.577
75	Octanoic acid	C06423	-0.577	1.155	-0.577
76	SDMA		-0.577	1.155	-0.577
77	Gly-Asp		-0.573	1.155	-0.581
78	*threo*-β-Methylaspartic acid	C03618	-0.568	1.155	-0.587
79	1-Methylhistidine<br>3-Methylhistidine	C01152	-0.549	1.154	-0.605
80	Adenylosuccinic acid	C03794	-0.506	1.152	-0.646
81	Trigonelline	C01004	-0.451	1.146	-0.695
82	Uridine	C00299	-0.447	1.146	-0.698
83	Pelargonic acid	C01601	-0.407	1.139	-0.732
84	UMP	C00105	-0.289	1.113	-0.824
85	Phosphorylcholine	C00588	-0.273	1.108	-0.835
86	Spermidine	C00315	-0.251	1.101	-0.851
87	UDP-N-acetylgalactosamine-2<br>UDP-N-acetylglucosamine-2	C00203<br>C00043	-0.250	1.101	-0.851
88	*N*-Acetylglucosamine 6-phosphate	C00357	-0.234	1.096	-0.862
89	Butyrylcarnitine	C02862	-0.225	1.093	-0.869
90	*N*-Acetylglucosamine 1-phosphate	C04256	-0.180	1.078	-0.898
91	Fumaric acid	C00122	-0.113	1.052	-0.938
92	Nicotinamide	C00153	-0.101	1.047	-0.946

93	GDP-glucose<br>GDP-mannose<br>GDP-galactose	C00394<br>C00096<br>C02280	0.022	0.989	-1.011
94	2,3-Diphosphoglyceric acid	C01159	0.071	0.963	-1.034
95	UDP-N-acetylgalactosamine-1<br>UDP-N-acetylglucosamine-1	C00203<br>C00043	0.230	0.865	-1.095
96	XA0065		0.286	0.826	-1.112
97	Hypoxanthine	C00262	0.378	0.756	-1.134
98	Triethanolamine	C06771	0.389	0.747	-1.136
99	Thiamine	C00378	0.392	0.744	-1.137
100	*S*-Adenosylmethionine	C00019	0.394	0.743	-1.137
101	Saccharopine	C00449	0.413	0.727	-1.140
102	Putrescine	C00134	0.473	0.676	-1.149
103	Ribulose 5-phosphate	C00199,C01101	0.490	0.660	-1.151
104	XC0001		0.496	0.655	-1.151
105	Cysteine glutathione disulfide	C05526	0.664	0.486	-1.150
106	UDP-glucuronic acid	C00167	0.688	0.459	-1.147
107	Gln	C00064,C00303,C00819	0.699	0.446	-1.145
108	GMP	C00144	0.705	0.439	-1.144
109	Stachydrine	C10172	0.712	0.431	-1.143
110	Malic acid	C00149,C00497,C00711	0.716	0.427	-1.143
111	5-Methyltetrahydrofolic acid	C00440	0.750	0.385	-1.135
112	3'-Dephospho CoA	C00882	0.768	0.362	-1.131
113	Trp	C00078,C00525,C00806	0.772	0.358	-1.130
114	ADP-ribose	C00301	0.785	0.341	-1.126
115	CDP-choline	C00307	0.787	0.338	-1.125

116	3-Hydroxybutyric acid	C01089,C03197	0.791	0.333	-1.124
117	2-Aminoisobutyric acid<br>2-Aminobutyric acid	C03665<br>C02261,C02356	0.801	0.319	-1.121
118	Aminoacetone	C01888	0.812	0.305	-1.117
119	Asn	C00152,C01905,C16438	0.819	0.296	-1.115
120	2-Hydroxybutyric acid	C05984	0.854	0.246	-1.100
121	Acetylcholine	C01996	0.859	0.238	-1.098
122	Glucuronic acid<br>Galacturonic acid	C00191<br>C00333	0.860	0.237	-1.097
123	*N*-Acetylglucosylamine	C01239	0.872	0.219	-1.091
124	Ala	C00041,C00133,C01401	0.882	0.204	-1.086
125	ADMA	C03626	0.889	0.193	-1.082
126	Lauric acid	C02679	0.890	0.192	-1.082
127	Thr-Asp		0.898	0.180	-1.078
128	Cystine	C00491,C01420	0.898	0.179	-1.078
129	AMP	C00020	0.907	0.165	-1.072
130	Fructose 6-phosphate	C05345,C00085	0.907	0.165	-1.072
131	Xanthine	C00385	0.914	0.154	-1.068
132	Glu-Glu	C01425	0.926	0.134	-1.060
133	β-Ala	C00099	0.940	0.111	-1.051
134	Fructose 1,6-diphosphate	C00354	0.944	0.104	-1.048
135	Glucose 1-phosphate	C00103	0.951	0.091	-1.042
136	*N*-Glycolylneuraminic acid	C03410	0.968	0.060	-1.029
137	Lactic acid	C00186,C00256,C01432	0.969	0.059	-1.028
138	ADP-glucose<br>GDP-fucose	C00498<br>C00325	0.971	0.055	-1.026
139	Glucose 6-phosphate	C00668,C01172,C00092	0.975	0.047	-1.023
140	XC0132		0.981	0.036	-1.018

141	Ascorbate 2-glucoside	C18339	0.987	0.026	-1.013
142	His	C00135,C00768,C06419	0.988	0.023	-1.011
143	N^6-Acetyllysine	C02727	0.992	0.016	-1.008
144	*N*-Acetyllysine	C12989	0.995	0.010	-1.005
145	*p*-Toluic acid<br>*m*-Toluic acid<br>*o*-Toluic acid	C01454<br>C07211<br>C07215	0.998	0.004	-1.002
146	*N*-Acetylaspartic acid	C01042	1.005	-0.010	-0.995
147	Hydroxyproline	C01157	1.014	-0.028	-0.986
148	3',5'-ADP	C00054	1.022	-0.046	-0.976
149	IMP	C00130	1.024	-0.049	-0.975
150	2-Hydroxyglutaric acid	C02630,C01087,C03196	1.025	-0.053	-0.972
151	Ser	C00065,C00716,C00740	1.028	-0.058	-0.970
152	5-Hydroxylysine	C16741	1.029	-0.061	-0.968
153	*N*-Acetylalanine		1.043	-0.091	-0.951
154	Theobromine	C07480	1.049	-0.107	-0.942
155	Glutathione (GSH)	C00051	1.053	-0.116	-0.937
156	Homocarnosine	C00884	1.058	-0.129	-0.929
157	Gly-Gly	C02037	1.063	-0.140	-0.923
158	*N*-Acetylmethionine	C02712	1.064	-0.145	-0.920
159	Isobutyric acid<br>Butyric acid	C02632<br>C00246	1.079	-0.184	-0.895
160	Sedoheptulose 7-phosphate	C05382	1.082	-0.192	-0.890
161	Citrulline	C00327	1.083	-0.196	-0.888
162	γ-Glu-Cys	C00669	1.084	-0.198	-0.886
163	PRPP	C00119	1.091	-0.219	-0.872
164	*N*-Acetylornithine	C00437	1.091	-0.219	-0.872

165	6-Phosphogluconic acid	C00345	1.093	-0.225	-0.869
166	Cys	C00097,C00736,C00793	1.096	-0.233	-0.863
167	Succinic acid	C00042	1.098	-0.238	-0.859
168	Uracil	C00106	1.098	-0.239	-0.859
169	Lys	C00047,C00739,C16440	1.103	-0.256	-0.847
170	Ophthalmic acid		1.104	-0.260	-0.845
171	Spermine	C00750	1.104	-0.261	-0.844
172	XC0061		1.113	-0.291	-0.822
173	*N*-Acetylgalactosamine<br>*N*-Acetylmannosamine<br>*N*-Acetylglucosamine	C01132<br>C00645<br>C00140	1.117	-0.306	-0.811
174	Tyr	C00082,C01536,C06420	1.119	-0.311	-0.807
175	4-Guanidinobutyric acid	C01035	1.121	-0.321	-0.800
176	Carnosine	C00386	1.127	-0.347	-0.780
177	Dyphylline	C07819	1.128	-0.350	-0.778
178	Argininosuccinic acid	C03406	1.128	-0.351	-0.777
179	Glycerophosphocholine	C00670	1.128	-0.352	-0.776
180	Met	C00073,C00855,C01733	1.129	-0.353	-0.775
181	Anserine_divalent	C01262	1.130	-0.358	-0.772
182	Leu	C00123,C01570,C16439	1.131	-0.365	-0.766
183	Daminozide<br>Ala-Ala	C10996<br>C00993	1.132	-0.370	-0.762
184	Thr	C00188,C00820	1.134	-0.378	-0.756
185	Ornithine	C00077,C00515,C01602	1.135	-0.381	-0.753
186	Gly	C00037	1.135	-0.385	-0.751
187	N^6,N^6,N^6-Trimethyllysine	C03793	1.136	-0.389	-0.747
188	Ascorbic acid	C00072	1.142	-0.423	-0.719
189	1-Methylnicotinamide	C02918	1.144	-0.438	-0.706
190	Cys-Gly	C01419	1.146	-0.448	-0.698
191	Glu	C00025,C00217,C00302	1.146	-0.449	-0.697

192	Phe	C00079,C02057,C02265	1.148	-0.466	-0.682
193	Guanidoacetic acid	C00581	1.150	-0.484	-0.666
194	Ile	C00407,C06418,C16434	1.151	-0.499	-0.652
195	Arg-Glu		1.152	-0.508	-0.644
196	Arg	C00062,C00792	1.155	-0.561	-0.594
197	*N*,*N*-Dimethylglycine	C01026	1.155	-0.573	-0.582
198	Isopropanolamine	C03194,C05771	1.155	-0.577	-0.577
199	Ergothioneine	C05570	1.155	-0.577	-0.577
200	Isocitric acid	C00311	1.155	-0.577	-0.577
201	Xanthosine	C01762	1.155	-0.577	-0.577
202	Trimethylamine	C00565	1.155	-0.577	-0.577
203	Trimethylamine *N*-oxide	C01104	1.154	-0.603	-0.552
204	Pro	C00148,C00763,C16435	1.154	-0.619	-0.535
205	Val	C00183,C06417,C16436	1.149	-0.672	-0.477
206	Methionine sulfoxide	C02989	1.148	-0.681	-0.467
207	5-Aminoimidazole-4-carboxamide ribotide	C04677	1.142	-0.717	-0.425
208	Hypotaurine	C00519	1.137	-0.744	-0.392
209	Terephthalic acid	C06337	1.136	-0.748	-0.388
210	Isovalerylcarnitine		1.134	-0.756	-0.377
211	*cis*-Aconitic acid	C00417	1.131	-0.768	-0.363
212	GDP	C00035	1.129	-0.773	-0.356
213	Tyr-Arg_divalent		1.122	-0.796	-0.326
214	Creatine	C00300	1.118	-0.810	-0.308
215	Myristoleic acid	C08322	1.057	-0.930	-0.127
216	Isobutyrylcarnitine		1.047	-0.946	-0.101
217	GABA	C00334	1.004	-0.996	-0.008
218	Gluconic acid	C00257	0.981	-1.018	0.038
219	*N*-Acetylglutamic acid	C00624	0.959	-1.037	0.078
220	Glycerol	C00116	0.709	-1.144	0.434

221	XC0089		0.701	-1.145	0.444
222	3-Indoxylsulfuric acid		0.636	-1.153	0.517
223	Hexanoic acid	C01585	0.623	-1.153	0.531
224	Isoglutamic acid	C05574	0.573	-1.155	0.582
225	Glycerol 3-phosphate	C00093	0.560	-1.155	0.595
226	GTP	C00044	0.513	-1.152	0.639
227	Sarcosine	C00213	0.491	-1.151	0.659
228	Serotonin	C00780	0.402	-1.138	0.737
229	Carboxymethyllysine		0.249	-1.101	0.852
230	Noradrenaline<br>6-Hydroxydopamine	C00547	0.244	-1.099	0.856
231	$NADP^+$	C00006	0.238	-1.097	0.860
232	Taurine	C00245	-0.008	-0.996	1.004
